# Supplementary figures and images for: The importance of large-diameter trees in the wet tropical rainforests of Australia
Source: PLoS One. 2019 May 1;14(5):e0208377. doi: 10.1371/journal.pone.0208377 (PMC6493708; doi:10.1371/journal.pone.0208377)

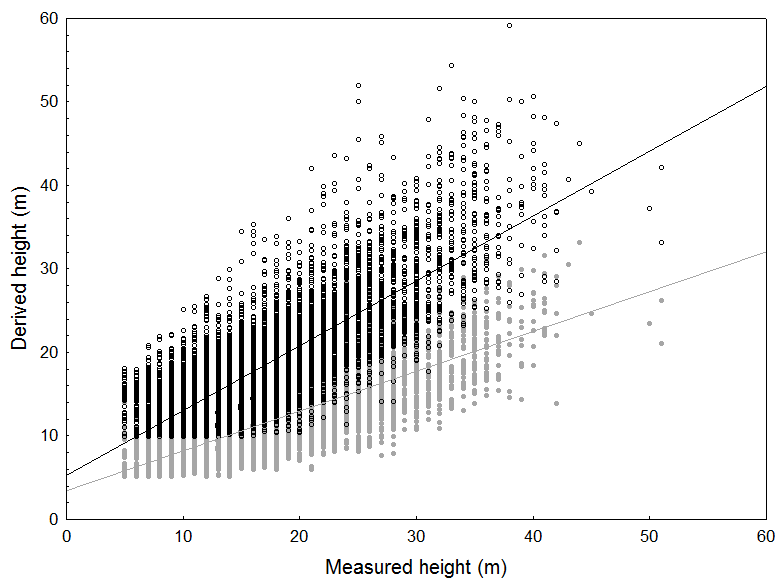

Supplement: S1 Fig — a) height derived from [29] equation 6a (grey circles; y = 3.444 + 0.477*x; r2 = 0.6285) and, b) height derived from [26] for Australian moist forest (back circles; y = 5.284 + 0.7766*x; r2 = 0.6293). (TIF) [file pone.0208377.s001.tif]

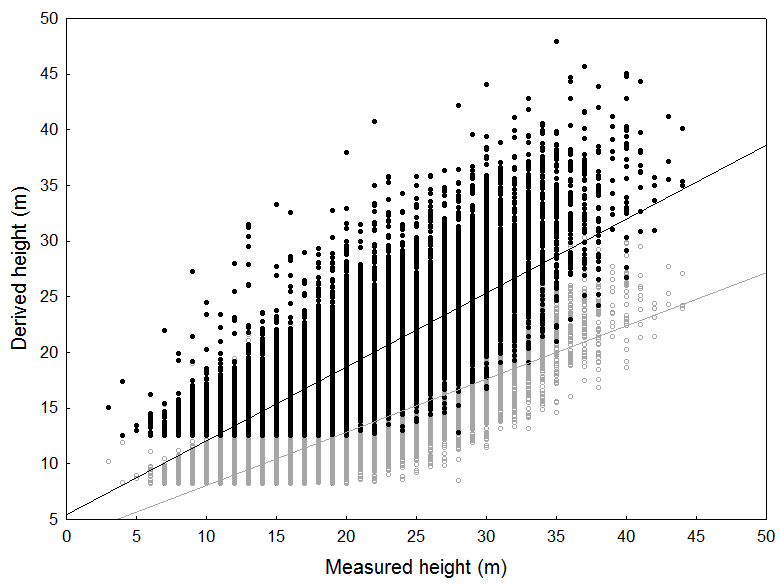

Supplement: S2 Fig — a) height derived from [29] equation 6a (grey circles; y = 3.2759 + 0.478*x; p = 0.0000; r2 = 0.6958 and, b) height derived from [26] for Australian moist forest (back circles; y = 5.4336 + 0.6638*x; p = 0.0000; r2 = 0.6866). (TIF) [file pone.0208377.s002.tif]

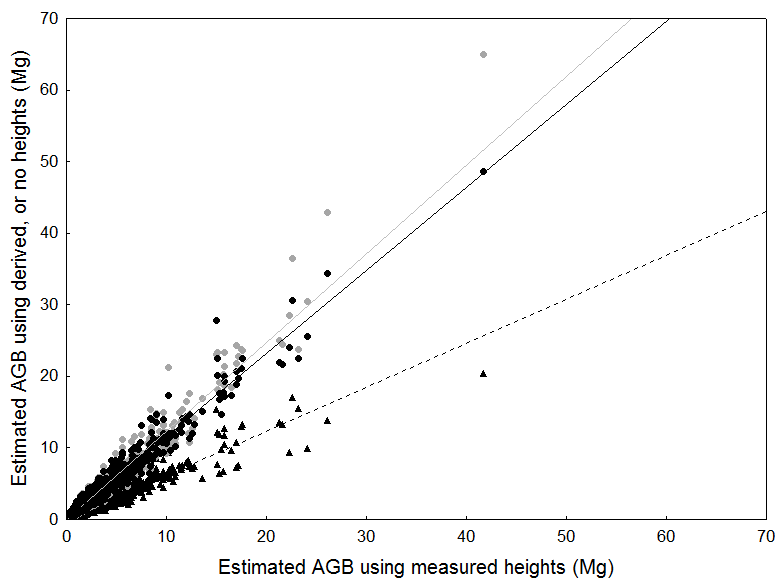

Supplement: S3 Fig — a) estimated AGB using [29] equation 2 with height derived from [29] equation 6a (triangles, dashed fit; y = 20.4771 + 0.6152*x; p = 0.0000; r2 = 0.9462), b) estimated AGB using [29] equation 2 using height derived from [26] for Australian moist forests (grey circles and fit; y = -59.9526 + 1.2396*x; r = 0.9793, p = 0.0000; r2 = 0.9590). c) estimated AGB using [28] for moist forests without height (black circles and fit; y = 1.2481 + 1.1607*x; r = 0.9852, p = 0.0000; r2 = 0.9706). (TIF) [file pone.0208377.s003.tif]
